# Characterization and Phylogenetic Analysis of *Campylobacter* Species Isolated from Paediatric Stool and Water Samples in the Northwest Province, South Africa

**DOI:** 10.3390/ijerph16122205

**Published:** 2019-06-21

**Authors:** Martina O. Chukwu, Akebe Luther King Abia, Eunice Ubomba-Jaswa, Lawrence Obi, John Barr Dewar

**Affiliations:** 1 Department of Life and Consumer science, College of Agriculture and Environmental Sciences, University of South Africa, Corner Christiaan De wet and Pioneer Avenue, 1724 Florida park Roodepoort, Gauteng 1709, South Africa; dewarj@unisa.ac.za; 2 Antimicrobial Research Unit, College of Health Sciences, University of KwaZulu-Natal, Private Bag X54001, Durban 4000, South Africa; lutherkinga@yahoo.fr; 3 Department of Biotechnology, University of Johannesburg, 37 Nind Street, Doornfontein, Gauteng 2094, South Africa; euniceubombajaswa@yahoo.com; 4 Water Research Commission, Lynnwood Bridge Office Park, Bloukrans Building, 4 Daventry Street, Lynnwood Manor, Pretoria 0081, South Africa; 5 Sefako Makgatho Health Science University, Molotlegi Street, Ga-Rankuwa, Pretoria, Gauteng, P.O Box 60, Medunsa 0204, South Africa; c355251@gmail.com

**Keywords:** *Campylobacter* spp., paediatric diarrhoea, antibiotic susceptibility profile, resistance genes, virulence genes, phylogenetic analysis, household drinking water

## Abstract

Antibiotic-resistant *Campylobacter* could adversely affect treatment outcomes, especially in children. We investigated the antibiotic susceptibility profiles, virulence potentials and genetic relatedness of *Campylobacter* spp. from paediatric and water samples in the North West Province, South Africa. Overall, 237 human and 20 water isolates were identified using culture and real-time polymerase chain reaction (PCR). The antibiotic susceptibility profiles were determined using the disk diffusion method. Gradient strips were used to determine the minimum inhibitory concentration of each antibiotic. Antibiotic resistance (*gryA*, *tetO* and *23S rRNA* 2075G and 2074C) and virulence (*cadF* and *ciaB*) genes were also investigated using PCR. A phylogenetic tree to ascertain the clonality between water and clinical isolates was constructed using MEGA 7. Overall, 95% (water) and 64.7% (human) of the isolates were resistant to at least one antibiotic tested. The highest resistance was against clarithromycin (95%) for water and ampicillin (60.7%) for human isolates. The *23S rRNA* 2075G/2074C mutation was the most expressed resistance gene. Phylogenetic reconstruction revealed eight intermixed clades within water and human *Campylobacter* isolates. This study suggests the possible circulation of potentially pathogenic antibiotic-resistant *Campylobacter* in the Northwest Province, South Africa with drinking water being a possible vector for disease transmission in this area.

## 1. Introduction

*Campylobacter* are small, spirally curved, Gram-negative, non-spore forming, bacteria with a single polar flagellum [1]. There are currently 32 species and 13 subspecies of the genus [2]. They are the most prevalent and frequent causes of food-related infections worldwide [1]. Their ability to multiply in an atmosphere that contains nearly 10% CO_2_ and 5% O_2_, with a temperature range of 30–46 °C, distinguishes them from other foodborne pathogens [3]. An increased incidence of infections due to *Campylobacter* has been noted both in developed and developing countries [1]. Most human cases present with gastroenteritis, which includes acute watery or bloody diarrhoea, abdominal pain, vomiting, fever and dehydration [1]. Amongst the known species of *Campylobacter*, *Campylobacter jejuni* is the most prevalent and frequently associated with diarrhoea or other bacteremic infections [4]. Other species such as *C. coli*, *C. lari* and *C. upsaliensis* have also been implicated in cases of gastroenteritis [1]. *Campylobacter* are highly pathogenic, capable of causing other health complications such as urinary tract infections, septicaemia, or some neuropathies including reactive arthritis, Guillain-Barre syndrome (GBS), irritable bowel and Miller Fisher syndrome (MFS) [1,5,6,7].

Molecular studies, using the whole genome sequence of *C. jejuni* NCTC 11168, have given insight into some of the essential virulence factors involved in the pathogenesis of *Campylobacter* infections [8,9,10]. These include the ability of *Campylobacter* to adhere (*pldA*, *cadF and capA* genes) and invade the intestinal epithelial cells (with the aid of the *CiaB* and *CiaC* genes), produce toxins (*cdtA* gene) and survive in the host cells [10]. In addition to pathogenesis, the increasing antibiotic resistance of *Campylobacter* isolated from humans, animals and the environment is fast becoming a significant public health concern [11,12,13]. Although gastroenteritis caused by *Campylobacter* is self-limiting, antibiotic treatment is advised in prolonged or bacteremic cases. Macrolides (erythromycin, azithromycin and clarithromycin), fluoroquinolones (ciprofloxacin) and tetracyclines, are the recommended antimicrobials for the treatment of infections due to *Campylobacter* [14]. However, resistance to these empirical drugs has been reported in many countries [12,15,16]. High-level tetracycline resistance is usually associated with the *tetO* gene, while mutation of the *gryA* or *parC* gene triggers resistance to fluoroquinolones [14,17]. Resistance to macrolides frequently occurs due to mutations at positions 2074 or 2075 of domain V in the *rrn* gene which encodes the *23S rRNA* gene [14].

Because *Campylobacter* spp. are isolated from a diverse host range, it is almost impossible to ascertain the source of human infection using culture and phenotypic characteristics. Molecular studies, such as phylogenetic analysis, have, however, helped in tracing the sources of clinical *Campylobacter* infections by exploiting differences in the genetic properties and frequency of *Campylobacter* strains that live in different hosts and environments [18]. Molecular methods have led to the detection of *Campylobacter* genes that are conserved within a given lineage and those that are phylogenetically distributed across the species [19]. They have also been used to detect similarity and differences in genes of particular strains isolated from the same host. For example, Gemmell et al. [20] used phylogenetic analysis to investigate the virulence properties and adaptive skills of *Campylobacter concisus (C. concisus)* isolated from the gastrointestinal tracts of humans and reported that there was no difference between oral and gut *C. concisus*.

*Campylobacter* infections are mostly contracted through the consumption of contaminated raw or undercooked poultry, unpasteurized milk or untreated water [21,22]. Although poultry is a known reservoir of *Campylobacter* spp., water has been reported to play a significant role in the transmission of *Campylobacter* infections, either directly (through drinking contaminated water or recreational use) or indirectly by colonizing livestock [23]. Waterborne *Campylobacter* outbreaks have been recorded in many countries [24]. Over 400 million cases of campylobacteriosis are recorded annually worldwide [1]. In Europe, up to 246,307 individuals were affected with campylobacteriosis in 2016 while an estimated one million people are infected yearly in the United States [2]. In Asia, the Middle East and Africa, *Campylobacter* infections are common, particularly in children [25,26].

Although studies have shown that *Campylobacter* infections could be acquired from water, those that report on the genetic relatedness of isolates from water and stool samples are few, especially in developing countries and South Africa. Establishing such relatedness could help identify points of intervention for the prevention of *Campylobacter*-related infections, particularly in resource-scarce settings. This study, therefore, (1) investigated the phenotypic and genotypic antibiotic susceptibility profile of *Campylobacter*, (2) ascertained the virulence capacity and the genetic relatedness of isolates of *Campylobacter* from paediatric samples and water samples collected from the North West Province, South Africa.

## 2. Materials and Methods

### 2.1. Ethical Statement

Approval to carry out the research was obtained from the College of Agriculture and Environmental Sciences (CAES) UNISA (2016/CAES/033), North-West Department of Health and Brits District Hospital. Written informed consent was obtained from parents/guardians of the participants, after which the participant’s age, gender, and clinical signs were recorded using a questionnaire before collecting the samples.

### 2.2. Stool and Water Sample Collection

Stool specimens were collected from September 2016 to December 2017. During this period, 505 fecal specimens were collected from diarrhoeal and non-diarrhoeal babies and toddlers under the age of 5 years that were attending the Brits District Hospital, Oukasie Clinic, Lethabeleng Clinic and Bopang Clinic in the Madibeng District of the North-West Province, South Africa.

A total of 92 water samples were aseptically collected from September 2016 to December 2017. These water samples were collected from different households that allowed the researcher access to the premises. The only inclusion criterion was that the household had a child that was less than five years old. A sample of stored water that was intended for drinking or cooking was collected from each household in a 5-L bottle by the researcher. A total of 88 water samples were collected from different homes of which eight were directly from a municipal tap and 80 from water storage containers. Of the stored water samples, 38 were fetched from the municipal tap while 42 were from a well/underground water or rainwater harvested from rooftops. Also, four samples were collected from the Crocodile River.

All collected water and stool samples were transported on ice to the Microbiology Laboratory of the Council for Scientific and Industrial Research (CSIR), Pretoria, for analysis.

### 2.3. Isolation of Campylobacter from Drinking Water and Fecal Materials

*Campylobacter* was isolated from stool samples using conventional methods that included the morphological appearance and catalase tests as previously published by Bessède et al. [27], with a little modification as discussed in Section 3.5. For the isolation of *Campylobacter* from water samples, methods described by Jokinen et al. [28] and Talay et al. [29] were used. Briefly, water samples were filtered through a 0.45µm sterile membrane in a vacuum filter. Membrane filters were placed in Bolton broth (BB) and incubated at 42 °C in a microaerophilic environment (MAE) for 24 h. After that, 100 µL of the enriched broth was placed onto a 0.6 µm membrane filter placed on tryptose blood agar (TBA) and incubated at 25 °C for 20 min. The membrane filters were then rolled out, and TBA plates were incubated at 42 °C in an MAE for 48 h.

### 2.4. Campylobacter Species Identification

Presumptive colonies from the culture plates were confirmed as being *Campylobacter* by targeting the genus-specific *16sRNA* gene using real-time PCR [30]. Real-time PCR was also used to confirm species using the primer *glyA* for *C. coli* and *C. upsaliensis* [31,32] and *hipO* for *C. jejuni* [32]. DNA was extracted using the heat lysis method [33]. The purity and concentration of the extracted DNA were determined spectrophotometrically using the Nanodrop Lite Spectrophotometer (Thermos Scientific, Waltham, MA, USA), and all samples had an *A* 260/280 ratio ranging from 1.7 to 2.1.

Real-time PCR was performed using a Corbett Life Science Rotor-Gene^TM^ 6000 Cycler (Qiagen, Hilden, Germany). The primers, 0.5 μL, (Forward and Reverse; final concentration 0.5 μM each), nuclease-free water (1 µL) and sample DNA (3 µL) were added to 10 µL of 2x SensiFAST^TM^ High-Resolution Melt (HRM) mix (SF) (Bioline GmbH, Luckenwalde, Germany), to obtain a final reaction volume of 15 μL. The cycling conditions included an initial activation at 95 °C for 10 min, followed by 40 cycles of denaturation at 95 °C for 10 s, annealing at 60 °C for 15 s and an extension at 72 °C for 20 s. The final step was an extension at 72 °C for 5 min. A melt curve was prepared by ramping up the melting temperature from 72 °C to 95 °C. Melt curve analysis was performed using the Rotor-Gene™ real-time analysis software, version 6.1 (build 93) (Corbett Life Science (Pty) Ltd., Sydney, Australia).

### 2.5. Antibiotic Susceptibility Testing

One hundred and fifty randomly selected confirmed clinical *Campylobacter* isolates (66 isolates of *C. jejuni*, 59 *C. coli* and 25 *C*. *upsaliensis*) and all confirmed water isolates (11 *C. jejuni*, 8 *C. coli* and 1 *C. upsaliensis*) were subjected to an antibiotic susceptibility test using the disc diffusion method [34,35]. These isolates were resuspended in sterile saline to attend a turbidity value equivalent to 0.5 McFarland. The suspension was inoculated onto a Mueller Hinton agar plate supplemented with 5% sheep blood. Assayed antibiotics: clarithromycin (15 μg/disc), erythromycin (15 μg), ciprofloxacin (5 μg), amikacin (30 μg), amoxicillin/clavulanic acid (30 μg), gentamicin (10 μg), ampicillin (2 μg), tetracycline (30 μg), cefuroxime (30 μg), cephazolin (30 μg), norfloxacin (10 μg), tigecycline (30 μg), meropenem (10 μg) and imipenem (10 μg) (DAVIES Diagnostics, Johannesburg, South Africa), were placed on the plates using a sterile forceps and incubated microaerophilically at 42 °C for 24 h. The inhibition zones were measured to the nearest millimeter using a ruler and interpreted according to reference values. The CLSI [35] and EUCAST recommended guidelines [36] breakpoints for macrolide was used for *C. jejuni* and *C. coli* while CLSI breakpoints for *Enterobacteriaceae* were used for other antibiotics. Quality control was achieved using *C. jejuni* (ATCC 33560) and *Escherichia coli* (ATCC 25922) [7,37].

### 2.6. Antibiotic Susceptibility Testing

The E-test strip method (Oxoid Ltd., Basingstoke, UK) was used to detect the MIC of the antibiotics [38]. The strip contained the required antibiotics at appropriate concentrations. The dilution range of the antimicrobial tested were: ciprofloxacin (32–0.015 µg/mL), erythromycin (256–0.015 µg/mL), tetracycline (256–0.015 µg/mL), ampicillin (256–0.015 µg/mL) and gentamycin (32–0.002 µg/mL). In brief, confirmed isolates from water and stool samples were grown on BA at 42 °C for 48 h. After incubation, a suspension was prepared in normal saline and adjusted to a 0.5 McFarland standard. The suspensions were spread onto a 5% sheep blood Muller Hinton agar using a sterile cotton swab, and then the antibiotic strips with different antibiotic concentration gradients were placed onto the agar plates and incubated for 24 h at 42 °C in a MAE. After the incubation, the MICs were measured, and the results were interpreted according to the National Committee for Clinical Laboratory Standards to *Enterobacteriaceae* [35,36].

The MIC was defined as the lowest concentration of an antibiotic that completely inhibited visible growth and was read at the point where the elliptical zone of inhibition intersected against the MIC scale on the strip. MIC_50_ and MIC_90_ were described in this study as the MICs that completely inhibited visible growth of 50% and 90% of the strains, respectively. The break-point criteria used for erythromycin and tetracycline were those of the CLSI for *Staphylococcus* species while for other drugs, criteria recommended for *Enterobacteriaceae* were used [39].

### 2.7. Detection of Antibiotic Resistance Genes

Genes conferring resistance to macrolides, fluoroquinolones, and tetracycline were screened on all water isolates and 206 clinical isolates, using the extracted DNA template. For the presence of quinolone resistance, the Thr-86-lle mutations that are found in the quinolone resistance-determining region (QRDR) of the *gyrA* gene in *Campylobacter* spp. was amplified [40]. The *tetO* gene responsible for tetracycline resistance was amplified, and for the presence of macrolides (erythromycin) resistance, point mutations were detected at position 2075 and 2074 in the *23S rRNA* gene [12,41] using specific primers that targeted the desired fragments (Table 1).

### 2.8. Sequence Assembly and Alignment

To determine the relatedness between the isolates, all isolates from water and 40 isolates from human samples were amplified using primers targeting the conserved region of the *16S rRNA* gene for identification of the *Campylobacter* genus [30]. The PCR products were purified and sequenced using Sanger sequencing at Inqaba Biotech^TM^ (Pretoria, South Africa). Sequence fragments were generated using Genetic Analyzer (Applied Biosystems, Foster City, CA, USA). A BLAST search was performed, and the sequences were compared to known *Campylobacter* sequences in the GenBank. After that the sequences were analysed, and a phylogenetic tree based on *16S rRNA* was created using MEGA7 [44,45].

## 3. Results

The participants in the current study included 257 males and 248 females. Of the 505 participants, 184 were on exclusive breastfeeding while 321 were on mixed feeding. Also, children with diarrhoea (321, with 82 having bloody diarrhoea) were more than those without (155). One hundred and fifty-nine participants reported vomiting while 209 had fever.

### 3.1. Detection of Campylobacter spp.

Overall, 108 *C. jejuni*, 89 *C. coli* and 40 *C. upsaliensis* were isolated from 505 paediatric diarrhoea and non-diarrhoea stool specimens. Of these, 81 *C. jejuni*, 78 *C. coli* and 29 *C. upsaliensis* were from diarrhoea and 27 *C. jejuni*, 11 *C. coli* and 11 *C. upsaliensis* were from non-diarrhoea stool samples. The detection of *Campylobacter* from individual water sources are shown in Table 2.

The overall *Campylobacter* recovery from water samples was 21.7% (20/92). The highest *Campylobacter* recovery (35.7%) was detected in well/harvested rainwater that was stored in a container. *Campylobacter* was not detected from river water and the water samples that were collected directly from the municipal tap.

### 3.2. Antimicrobial Susceptibility of Campylobacter Isolates

Of the 14 antibiotics tested, the highest phenotypic resistance displayed by *Campylobacter* isolates from water samples was against clarithromycin (95%), while complete susceptibility (100%) was observed against imipenem. From the human samples, the highest resistance was observed against amoxicillin/clavulanic acid (64.7%), while resistance to imipenem was the least observed (15.3%) Table 3.

The distribution of antibiotic resistance according to species showed different patterns (Table 4) with resistance ranging from 13.3% to 95%.

### 3.3. Determination of the Minimum Inhibitory Concentration (MIC)

The MICs of the five antibiotics tested are shown in Table 5. All isolates were highly resistant to ampicillin. *C. upsaliensis* isolates from human samples were susceptible to ciprofloxacin and gentamicin while the isolate from water was only susceptible to erythromycin.

### 3.4. Prevalence of Multiple-Antibiotics Resistance (MAR)

**Figure 1:**
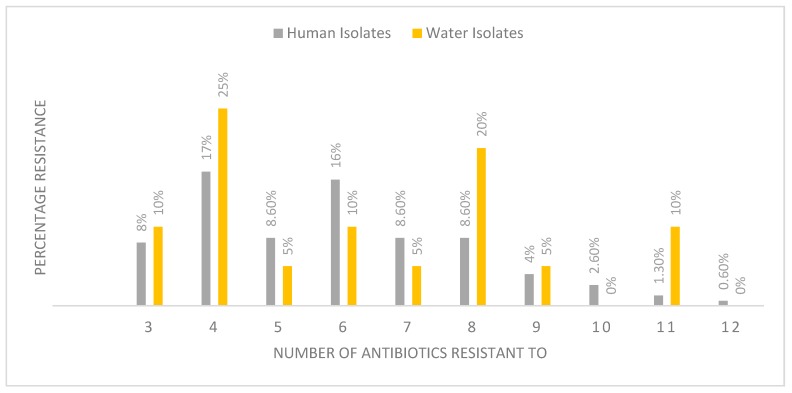
The overall percentage of multi-antibiotic resistance of *Campylobacter* isolates.

The overall prevalence of MAR revealed that 76% stool and 90% water *Campylobacter* isolates were simultaneously resistant to more than three of the antibiotics tested (Figure 1). MAR in this study was defined as the resistance of *Campylobacter* to two or more antibiotics [13]. Two isolates each from water and human stools were concurrently resistant to up to 11 antibiotics. The presence of MAR was observed more in *C. jejuni* isolates.

### 3.5. Expression of antibiotic resistance genes by Campylobacter isolates

Table 6 shows the distributions of the antibiotic resistance genes among the species; 28.6% of the human isolates harboured one or more genes, while more than 90% was found from the water samples.

The *tetO* gene was the most amplified gene. It was found in 59/206 (28.6%) of the human samples tested (45 isolates that were simultaneously resistant to tetracycline and tigecycline). Also, 14 of those that did not undergo a susceptibility test expressed the *tetO* gene.

All the isolates phenotypically resistant to tigecycline harboured the *tetO* gene. Also, 45/48 (94%) of the *human* isolates that were resistant to tetracycline expressed the *tetO* gene. Among the species, the *tetO* gene was found more in *C. jejuni* (31.8%; 29/91) compared to *C. coli* 30.8% (25/81) and *C. upsaliensis* 14.7% (5/34). From water samples, the *tetO* gene was found in 40% (8/20) of the isolates, and all the isolates that expressed this gene were phenotypically resistant to tetracycline. At the species level, the *tetO* gene was found more in *C. jejuni* isolated in human samples, while in water samples it was amplified more in *C. coli* isolates.

Transitional mutations at position A2075 and A2074 in the V region of the *23S rRNA* gene was the most amplified gene in the water isolates, while from the human samples it was the second most amplified gene. From the water samples, 85% of the isolates (17/20) that were phenotypically resistant to erythromycin had an A2074 point mutation, while 16/20 (80%) isolates that were resistant to both erythromycin and clarithromycin showed mutations at position A2074 and A2075 in the V region of the 23S rRNA. From clinical strains, mutations at A2075 occurred in 19.4% (40/206) of the isolates, while a mutation at position A2074 was found in 18.4% (38/206) of the isolates. Also, 18.4% (38/206) of the isolates that were concurrently resistant to the macrolides tested expressed mutations at both positions (A2074/2075) of the V region. Furthermore, two human isolates that were susceptible to erythromycin but resistant to clarithromycin also expressed a mutation at position A2075. *C. coli* isolates 24.6% (20/81) expressed the highest mutation rate compared to *C. jejuni* 18.6% (17/91).

The quinolone resistance-determining region (QRDR) of the *gryA* gene was amplified in 18.4% (38/206) of the clinical isolates and 25% (5/20) of the water isolates. All strains were concurrently resistant to ciprofloxacin and norfloxacin, that is, all fluoroquinolone-resistant isolates expressed the *gryA* gene. Also, 11 isolates that were excluded during the susceptibility test also expressed the *gryA* gene. Distribution of the *gryA* gene according to species is shown in Table 6. Isolates haboring multiple resistance genes were also observed in the clinical as well as the water samples. Thus, from the clinical setting, *tetO* and *gryA* genes were found in 6.8% (14/206) of the isolates. The *tetO* gene and mutation at position A2075G/ A2074C were found in 9.7% (20/206) of the samples. Of these, 12% (11/91) were *C. jejuni*, 8.6% (7/81) *C. coli* and 5% *C. upsaliensis*. *C. upsaliensis* identified in the water samples harboured all the resistance genes tested. Sequenced samples all showed similarities with known *gryA* and *tetO* genes of *Campylobacter jejuni* and *Campylobacter coli* in the GenBank.

### 3.6. Expression of Virulence Genes among Campylobacter Species

The result of the virulence genes showed that 85% of the water harboured the *ciaB* and *cadF* gene, while from human samples, *ciaB* was expressed in 38% and *cadF* in 51% of the isolates (Table 7). From the human samples, the *ciaB* gene was expressed more in *C. coli* isolates (39.3%), while the *cadF* gene was found more in *C. jejuni* (54.6%). A combination of the *ciaB* and *cadF* genes was found in 24% of the isolates, of which 22.2% (24/108) of the combinations were found in *C. jejuni*, 23.5% (21/89) in *C. coli* strains and 30% (12/40) in *C. upsaliensis*. From the water samples, 90.9% of *C. jejuni* expressed the *cadF* gene.

Comparative analysis of the demographics showed that there was an interaction between the virulence genes and *Campylobacter* infected diarrhoea cases. Thus, out of the 90 human isolates that expressed the *ciaB* gene, 75 (83%) were from diarrhoeal cases. Also, 124 of the 130 (95.3%) isolates that carried the *cadF* gene were from diarrhoeal cases. The virulence genes were also expressed in 61.7% of children that had a fever and 51.7% that reported vomiting. Also, stool samples from male children harbour more virulence genes; *ciaB* 50/90 (55%) and *cadF* 66/130 (50.7%) compared to samples from females, *ciaB* 40/90 (44.4%) and *cadF* 64/130 (49.2%). Samples of children on mixed feeding expressed more virulence genes, *ciaB*, 58.8% (53/90), *cadF* 67.7% (88/130) than those on exclusive breastfeeding, *ciaB* 41% (37/90) *cadF* 36% (47/130).

### 3.7. Phylogenetic Relationship of Campylobacter Strains by Partial Genome Sequencing

**Figure 2:**
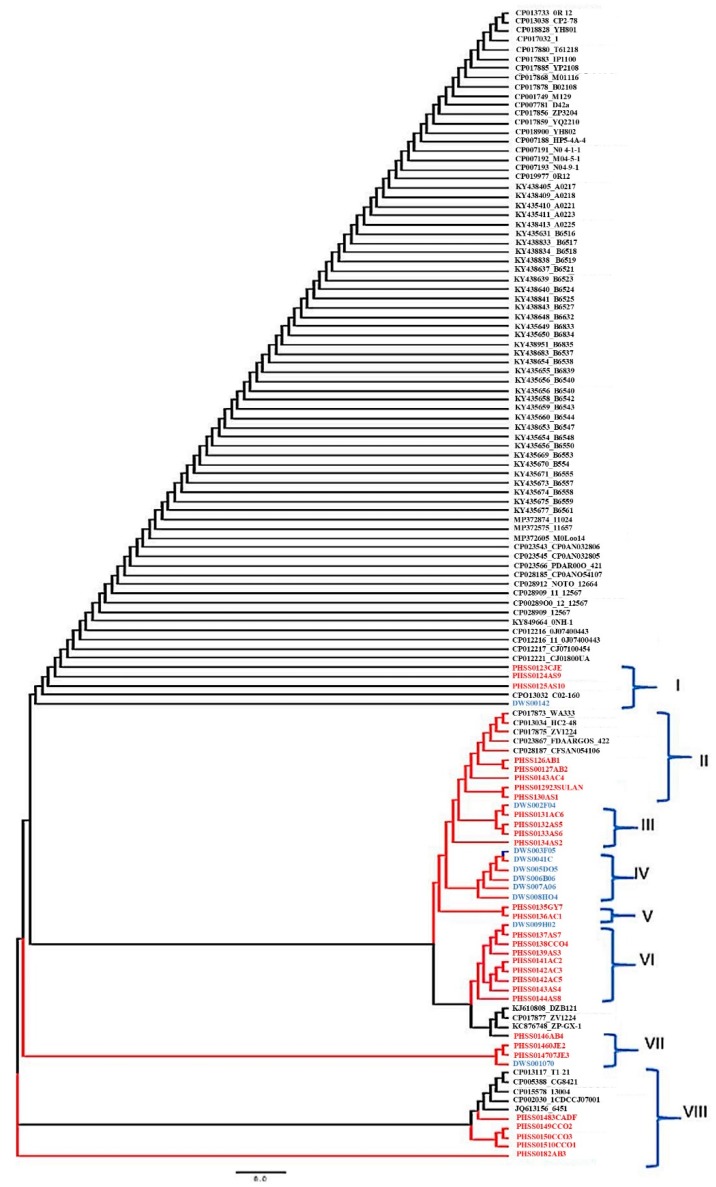
Phylogenetic tree displaying relatedness between human and water isolates.

Phylogenetic reconstruction revealed five different clades. These clades were placed into eight groups (Groups I–VIII) according to how closely related the strains were. Groups I, III, VI and VII contained the sequences that were intermixed with *Campylobacter* spp. isolated from both human and water samples (Figure 2). Groups II, V and VIII, consisted of *Campylobacter* strains that were circulating within the studied human population. Group IV were strains that only existed in water.

## 4. Discussion

*Campylobacter* spp. are identified as etiologic agents in outbreaks and sporadic cases of diarrhoea and gastrointestinal infections worldwide. A recent report by the Global Enteric Multicentre Study group (GEMS) indicated *Campylobacter* as one of the primary agents that causes diarrhoea in developing countries [46], and infection is usually limited to children [47,48]. Untreated drinking water has been noted as a significant source of *Campylobacter* infections and outbreak [22]. This study, therefore, investigated the genetic similarity of 257 *Campylobacter* strains isolated from paediatric stool and household drinking water samples in the Northwest Province of South Africa. Overall, 119 *C. jejuni* (108 from paediatric stools and 11 from water), 97 *C. coli* (89 strains from stools and 8 from water) and 41 isolates of *C. upsaliensis* (40 from stools and 1 strain from water) were isolated and screened against 14 different antibiotics. Human isolates exhibited different levels of resistance against all the antibiotics tested, while some water isolates were simultaneously resistant to up to 13 different antibiotics. The *23S rRNA 2075G/2074C* mutation and *tetO* gene were the most expressed of all the resistance genes analysed. Phylogenetic reconstruction revealed eight clades that were intermixed within *Campylobacter* spp. isolated from both water and human samples.

### 4.1. Detection of Campylobacter spp.

Clinical *Campylobacter* species were isolated from paediatric patients with diarrhoea and those without diarrhoea, while water isolates were from stored household water and municipal tap water sources. Twenty-one percent of the water samples were contaminated with diverse *Campylobacter* species. Several studies have assessed the prevalence of *Campylobacter* spp in different water sources [1,49,50,51]. In South Africa, 13% was reported in surface and groundwater [52], and in New Zealand, 75% and 29.2% were found in groundwater and drinking water, respectively [53]. The contributions of water to the burden of sporadic cases of *Campylobacter* infections might be unknown because not all cases lead to severe illness and most often a milder degree of illness might not require medical attention [22]. As such, infected people might not report to a hospital, thus affecting the overall prevalence within a given community. However, most outbreaks have been mainly attributed to the drinking of contaminated water [22], indicating the role water plays in the transmission of *Campylobacter* infections.

No *Campylobacter* contamination was recorded in the water samples collected from the Crocodile River. These results were surprising considering that river water has been reported as a reservoir of *Campylobacter* spp. and prevalence ranging from 60% to 79% have been found [53,54]. However, it should be noted that *Campylobacters* have been reported as non-indigenous to aquatic environments, mainly because of their growth requirements, and their presence is indicative of recent faecal contamination [55]. Hellein et al. [56] reported that aquatic *Campylobacter* contamination reflected sewage effluent contamination and agricultural runoff. Thus, it could be assumed that the inability to isolate *Campylobacter* from the river water sample might be associated with the choice of site because the river water samples analysed in this study were collected from areas that were less impacted by human activities. *Campylobacters*, like many other enteric pathogens, do not occur in high concentrations in aquatic environments, so isolation usually requires the concentration of larger volumes of water, particular growth requirements and more extended incubation periods; in the absence of the above-mentioned conditions, results in most cases are usually false negatives [57]. Also, it has been reported that *Campylobacters* can enter the viable but non-culturable (VBNC) state when exposed to prolonged poor nutrient and unfavourable temperature conditions in aquatic environments [52]. These factors, coupled with the few numbers of samples included in the current study, could have affected the results obtained.

Most *Campylobacter* outbreaks usually highlight infections emanating from inadequate disinfection and filtration or sewage contamination and drinking contaminated water is the most accepted cause of *Campylobacter* enteritis outbreaks [22,52]. In the present study, water samples collected directly from the municipal taps were all negative for *Campylobacter*, indicating that municipal taps might not be the source of water contamination. It could also mean that treatment at the waterworks could effectively remove *Campylobacter* from raw water, as studies have reported on the sensitivity of *Campylobacter* strains to disinfection [58,59]. The highest *Campylobacter* contamination in the present study was found in water samples collected from stored containers (rainwater, well and municipal taps). Several factors have been linked to the poor microbial quality of stored household water [50]. Although treated municipal water may contain residual chlorine to ensure the safety of the water during storage, prolonged storage within the house could lead to recontamination of the water. The type of container may also allow for recontamination of previously treated water [50,60]. For example, wide open-neck containers would allow for recontamination during extraction of the water using dirty containers. Another critical factor is the source of water. Most of the samples analysed in the current study were collected from wells or rain. These water sources have been reported to contain substantial numbers of microorganisms, including pathogenic ones [60,61]. Thus, storing such water in the household without pre-treatment could favour the growth of the already present microorganisms.

### 4.2. Antibiotic Resistance Profiles of Campylobacter Species

Antibiotic resistance has been documented as a global pressing public health concern. In developing countries, the situation is deteriorating more rapidly because of the widespread and uncontrolled use of antimicrobial agents [13,62]. Although *Campylobacter* infections are self-limiting, antibiotics may be prescribed to patients with unusually severe and prolonged symptoms or in immunocompromised patients [14]. In recent years, antibiotic resistance (ABR) has been reported in some *Campylobacter* spp. in most countries [47]. In the present study, 150 isolates from paediatric stool samples and 20 isolates from domestic water samples were tested against 14 antibiotics. About 65% of clinical isolates and 95% of isolates from the water samples were resistant to at least one antibiotic tested. In previous reports, antibiotics in the carbapenem group have shown an excellent in vitro activity against *Campylobacter* spp. [63,64]. Correspondingly, in this study, a low antibiotic resistance was observed to imipenem and meropenem. A relatively low resistance was also observed against gentamicin among the clinical isolates although gentamicin has been reported by the WHO as an alternative in cases of sepsis and some neonatal bacteraemia [65]. However, the prolonged use of gentamicin can lead to renal tubular dysfunction in children [66]. Compared to the water isolates, all clinical isolates showed the highest resistance to macrolides and penicillin with resistance rates of 80% and 85% respectively. Minimal resistance was experienced against other antibiotics except imipenem with 100% susceptibility.

Based on the results of this study, no currently tested antibiotic reliably covered all the clinical *Campylobacter* strains identified in this study. Among the empirical drugs, antibiotic resistance was slightly lower against the fluoroquinolones. Norfloxacin exhibited a lower resistance (13.3%) and offered a better alternative than ciprofloxacin (18%). Fluoroquinolones are among the recommended drugs for the treatment of campylobacteriosis [16]. However, treatment with fluoroquinolones has become quite a challenge as some *Campylobacter* spp. have developed resistance to this class of antibiotics [67]. Infections due to fluoroquinolone-resistant *Campylobacter* strains are usually severe and last longer. Fluoroquinolone-resistant *Campylobacter* have been reported to be responsible for 23% of all campylobacteriosis in the United States and cause an estimated 310,000 illnesses per year [68]. In *Campylobacter* and other Gram-negative bacteria, the fluoroquinolones act by inhibiting the function of topoisomerase enzymes (topoisomerase II & IV) and DNA gyrase. Studies conducted in many countries have shown that alteration or mutation in the gyrase A (*gyrA* gene) of Gram-negative bacteria might result in an automatic resistance to fluoroquinolones [13,14]. The percentage resistance recorded for the clinical and water isolates against ciprofloxacin and norfloxacin agrees with a previous prevalence report in South Africa, where *Campylobacter* resistance rates to fluoroquinolones between 14.8% and 51.3% were recorded [69]. These results illustrate that *Campylobacter* resistance to fluoroquinolones might not have increased over the years. However, constant monitoring is required as *Campylobacter* spp. can mutate [70]. Antibiotic resistance prevalence among the species showed that clinical *C. jejuni* isolates were highly resistant to ciprofloxacin (24.2%) and norfloxacin (16.6%) compared to *C. coli* (18.6%) and (8.4%), respectively, contrary to the pattern observed with the water isolates. Only one isolate of *C. upsaliensis* was identified from the water samples, and the strain was resistant to fluoroquinolones. Also, from the clinical specimens, 4% of the *C. upsaliensis* was resistant to norfloxacin. A previous study in Denmark recorded resistance to fluoroquinolone ranging from 48.2% (*C. jejuni*) to 66.7% (*C. coli*) [71]. Similarly, 42% of *C. jejuni* and 83% of *C. coli* isolates, recovered from patients with travelers were resistant to fluoroquinolones in a study conducted in Finland [72], while 63.2% of resistance to fluoroquinolones has been reported in patients with severe diarrhoea in the United Arab Emirates [11]. Previous reports have speculated that the spread of fluoroquinolone resistance in human isolates might have originated from the excessive use of veterinary fluoroquinolones (enrofloxacin and danofloxacin) in food-producing animals [73]. For example, in Australia, where the use of fluoroquinolone is banned in food-producing animals, a rate of 0–2% resistance was reported [74]. However, antibiotic resistance of *Campylobacter* isolates in food-producing animals has not been studied in many developing countries, including South Africa. Thus, conducting similar studies on poultry, for example, would enhance the understanding of possible sources of antibiotic resistance in the study locality.

Given the increasingly high incidence of fluoroquinolone resistance in *Campylobacter* spp., macrolides were considered the alternative drugs of choice for the treatment of human campylobacteriosis [67]. The incidence of *Campylobacter* resistance to macrolides in clinical isolates was previously rare, especially in developed countries [41]. Recent reports from different parts of the world have, however, shown that *Campylobacter* spp. have acquired resistance to this class of antibiotics. The isolates in the current study showed varying resistance rates against the macrolides tested (erythromycin; 26.7% and clarithromycin; 29.3%). Previous studies in South Africa have reported up to 53% resistance to macrolides [12,69], while a resistance of 86.1% has been reported in India [13]. Studies from other parts of the world have reported that *C. coli* strains often show increased resistance to macrolides compared to *C. jejuni* [72]. However, C. *jejuni* was the most resistant to macrolides compared to other species in the current study. A previous study in South Africa recorded resistance to ciprofloxacin and erythromycin in 33.3% and 38.9% of *C. coli* and 20% and 31.5% of *C. jejuni*, respectively [12]. An increase in resistance to both erythromycin and ciprofloxacin has also been documented in other developing countries [47], and it is speculated to be primarily influenced by the use of macrolides for infections other than gastrointestinal diseases and the pressing issue of self-medication [75,76]. It might also be due to horizontal transfer of resistance genes from animals to humans as it has been documented that macrolides like spiramycin, erythromycin and tylosin prevent infection in animals or act as growth promoters [67,75]. Reports from the Centers for Disease Control and Prevention (CDC) showed that macrolide-resistant *Campylobacter* strains are responsible for 2% of campylobacteriosis in the United States and cause an estimated 22,000 illnesses and up to 5 deaths annually [68]. Thus, the high resistance to macrolides recorded in the current study calls for more stringent measures to prevent the spread of these bacteria, especially within settings with limited resources such as the North West Province. Also, the higher prevalence of macrolide resistance recorded in water compared to the clinical isolates is a call for concern, given that the consumption of untreated contaminated water has been linked with numerous waterborne *Campylobacter* disease outbreaks around the world.

Increase in tetracycline resistance has been reported to emerge from the extensive use of these antibiotics as prophylaxis and therapy of human and animal infections and in promoting animal growth [77]. Thus, *Campylobacter* resistance to tetracycline has been frequently reported in humans, animals and aquatic environments [78,79,80]. The current study observed a resistance rate of 32% of clinical *Campylobacter* isolates to tetracycline and 55% from water isolates. Among the species, *C. jejuni* strains from human samples showed a higher resistance to tetracycline compared to other strains, while from water samples, resistance to tetracycline was observed more among *C. coli* isolates. A study in Quebec, Canada, reported a 50% tetracycline resistance among *C. coli* isolates and 39% among *C. jejuni* isolates [78]. In Spain, *C. coli* (94%) and *C. jejuni* (36%) isolated from water samples were reported to be resistant to tetracycline [80]. Moreover, in South Africa, 55% resistance among *C. coli* isolates and 25.9% among *C. jejuni* isolates have been reported [12]. Contrarily, C. *jejuni* isolates showed the highest resistance in the current study compared to the other species. The resistance of *Campylobacter* spp. exhibited against tetracyclines in the current study suggests a potentially high risk of treatment failure in *Campylobacter* infections and highlights the importance of monitoring antibiotics and the quest for alternative strategies to treat bacterial infections.

### 4.3. Multi-Antibiotic Resistance (MAR)

There is an increased trend in the occurrence of MAR pathogenic organisms worldwide [81]. MAR, especially to macrolides, fluoroquinolones and tetracyclines, is a considerable concern, and it is considered highly undesirable in *Campylobacter* as these three classes are generally advocated for as the first-line drugs for the treatment of *Campylobacter* enteritis [35]. The first ever reported case of MAR *Campylobacter* in South Africa was observed in children less than five years old at the Red Cross Children’s Hospital in Cape Town [82]. Since then, MAR *Campylobacter* strains have been frequently encountered in human, animals and water samples [79,83]. MAR *Campylobacter* strains were believed to emerge from the consumption of poultry meat because antibiotics are used in poultry production as growth promoters [72,84]. However, studies have shown that the excessive use of antibiotics by humans can also lead to the development of MAR in human isolates [62]. The present study recorded MAR in 76% and 90% of clinical and water isolates, respectively. These isolates were resistant to more than three antibiotics agents. All three *Campylobacter* species analysed in the current study exhibited MAR. However, the highest resistance observed from the clinical samples was found in *C. upsaliensis* (94.4%). In Qatar, United Arab Emirates, 6.3% of MAR *Campylobacter* strains were isolated from patients with bloody diarrhoea [11]. MAR strains of C. *jejuni* (52.6%) and *C. coli* (47.4%) recovered from Finish patients were found to be co-resistant to tetracycline and amoxicillin/clavulanic acid [72]. In Ghana, 100% MAR was recorded in water samples [81]. Resistance to relevant therapeutic agents poses a risk when there is no effective antimicrobial regimen for *Campylobacter* infection making treatment unattainable. Studies have shown that patients infected with MAR *Campylobacter* strains have an increased risk of an adverse event compared to patients infected with a susceptible *Campylobacter* strain [71].

### 4.4. Distribution of Antibiotic Resistance Genes

Antibiotics in the tetracycline family act by inhibiting bacterial protein synthesis. They achieve this by preventing the attachment of aminoacyl-tRNA to the ribosomal acceptor (A) site [60,62,85]. Resistance to tetracyclines in most bacteria is often due to the acquisition of new conjugate genes that are associated with plasmids or transposons [77]. In *Campylobacter*, the principal determinant of tetracycline resistance is a plasmid-borne gene, belonging to the tet family of proteins (tetO), which confers resistance by displacing tetracycline from its primary binding site on the ribosome [62]. Studies have shown that tetO protein reduces the susceptibility of ribosomes to the action of tetracyclines when guanosine triphosphate (GTP) is present [62]. The *tetO* gene was the most amplified resistance gene in the human samples and the second most amplified gene in water isolates in the current study. All the human isolates that were phenotypically resistant to tetracycline harboured the *tetO* gene, while only 40% out of the 50% of the water isolates that were phenotypically resistant to tetracycline expressed the *tetO* gene. The prevalence of the *tetO* gene in C. *jejuni* strains was higher compared to other species. It should be noted that *C. jejuni* isolates in this study were mostly from paediatric patients with severe clinical symptoms, suggesting that the resistant strains could have contributed to the severity of the infections caused. *C. coli* and *C. upsaliensis* also expressed the *tetO* gene at 20% (25/89) and 12% (5/40), respectively. In Canada, a higher prevalence of the *tetO* gene has also been reported among human isolates [78]. High prevalence of the *tetO* gene poses a threat in the treatment of campylobacteriosis given that the *tetO* gene can be transferred rapidly from a resistant isolate to a susceptible isolate [85].

Just like tetracyclines, macrolides also inhibit bacterial growth by binding to the bacterial ribosomes and interfering with protein synthesis. In *Campylobacter*, macrolide resistance is chromosomally mediated and is associated with a reduction in macrolide binding affinity to the ribosomal 23S subunit [86]. Thus, *Campylobacter* spp. may evade macrolides by altering the antibiotic’s target site at the V region of the *23S rRNA*. Most often, alterations at position 2074C or 2075G proffer high-level resistance. It has been reported that *Campylobacter* strains carrying these mutations are stable in culture, and also maintain their ability to colonize their host [63]. In the current study, 55 out of the 62 human isolates (88.7%) analysed and 15 out of the 17 water isolates (88.2%) had this mutation. These isolates had very high MICs and expressed both mutations at A2074C and A2075G. Isolates that were either resistant to clarithromycin or erythromycin had a mutation at position A2075G only. *Campylobacter* isolates that express mutations at position A2075G and A2074C extend high-level resistance [87]. The findings of the current study corroborate the report of Vacher et al. [88] who observed point mutations at position A2074C and A2075G in the *23S rRNA* gene in 99.3% of *C. coli* and *C. jejuni* isolates in their study [88]. Similar to the present study, single mutations at positions A2075G or A2074C have also been reported previously [14]. Also, combined mutations at A2075G and A2074C have been reported to confer a higher level of erythromycin resistance among *Campylobacter* isolates [67,87]. Previous studies reported that resistance in *C. coli* strains was associated with a mutation at position A2075G and A2074C [87]. This report agrees with the present study as the rate of expression of mutations at position A2075G/ A2074C was higher in *C. coli* isolates compared to *C. jejuni* and *C. upsaliensis*.

Members of quinolone antibiotics target two large bacterial enzymes, the DNA gyrase and the topoisomerase IV. Studies have shown that binding of quinolones to these enzymes inhibits the synthesis of bacterial DNA, which ultimately causes cell death [39,89]. However, some bacteria have developed resistance to this set of antibiotics by substituting amino acids at the quinolone resistance-determining region (QRDR) of the topoisomerase [3]. In *Campylobacter*, resistance to quinolones is primarily mediated by a single point mutation in the QRDR of the *gyrA* gene at codon 86 (that is, an alteration of the nucleotide from ACA to ATA), leading to isoleucine substitution for threonine [90,91]. Although there are different types of amino acid substitutions, the most frequently observed is the C_257_T mutation in the *gyrA* gene which leads to Thr86Ile substitution in the gyrase and confers a high level of resistance to this class of antibiotics [90]. In the current study, 18.4% of the human and 25% of the water *Campylobacter* isolates harboured the *gryA* gene. The prevalence of the *gryA* gene was higher in *C. upsaliensis*, and *C. jejuni* compared to *C. coli*. Previous studies have reported a similarly high occurrence of the *gryA* gene in *C. jejuni* strains. For example, a study conducted in Europe reported that over 41% of *C. jejuni* isolated from humans, poultry products, water and wild bird carcasses expressed the *gyrA* gene [90]. Pere-boto et al. also reported that the *gyrA* gene is the most prevalent resistance gene in clinical isolates collected from 10 different provinces in Spain, and was expressed mostly in *C. jejuni* isolates that exhibited high ciprofloxacin MIC [14]. Previous studies have reported that most ciprofloxacin-resistant *Campylobacter* spp., especially *C. jejuni* strains, express the Thr86Ile amino acid substitution in the QRDR of *gyrA* [40,90,92]. The CDC, in 2013, reported that fluoroquinolone resistance among *Campylobacter* strains comes with a heavy economic burden because infections caused by fluoroquinolone-resistant strains stay longer and can in most cases lead to death [68].

### 4.5. Detection of Campylobacter Virulence-Associated Genes

The mechanism by which *Campylobacter* causes human diseases is believed to be multifactorial [10]. Specific genes involved in adhesion, colonisation, invasion and toxin production are necessary for the process of infection [93]. To determine the pathogenic potentials of the *Campylobacter* isolates in the current study, the presence of two essential genes coding for virulence determinants such as the adhesive (*cadF*) and invasive (*ciaB*) genes in the isolates was investigated. These virulence factors were expressed more in water isolates compared to clinical isolates. The higher prevalence of virulence genes noticed among the water isolates in this study contradicts other published studies in which relatively higher number of virulence genes were identified in human clinical samples [94,95]. These results, therefore, indicate that the *Campylobacter* spp. isolated from water samples in this study might be highly virulent, and could attach and invade the host epithelial cells [43].

Interestingly, not all diarrhoeal isolates in the present study expressed the virulence genes. Given that the *cadF* gene aids *Campylobacter* to adhere to the host gastrointestinal epithelium and for internal colonization [95], while the *ciaB* gene is required for maximal invasion of intestinal epithelial cells [96], it would be expected that all isolates identified from diarrhoea cases would harbour the virulence genes. It has been previously reported that *Campylobacter* strains that lack the *cadF* gene were unable to colonise in chicken models and their internalisation ability was compromised [97]. The results of the current study are in agreement with those of Koolman et al. who tested for the presence of a series of virulence genes in *Campylobacter* isolates and observed that not all strains possessed adhesin proteins and that some strains that possessed the genes could invade Caco-2 cells [93]. Similar reports had earlier been published by Ziprin et al. [43], where the *C. jejuni cadF* mutants were unable to colonise chickens. It has been suggested that the lack of the *cadF* gene and inability to colonise and bind intimately to the host cells as exhibited by some *Campylobacter* strains is due to their inability to overcome different biological barriers and stressors encountered in the host cell, including increased temperature of the host and the acidity level of the stomach [10,43].

The low prevalence of the *cadF* and *ciaB* genes in the clinical isolates, however, contradicts other previously published data reporting 100% detection of these virulence determinants in their isolates [42,95,98,99]. The results of the present study, therefore, confirm the argument that not all *Campylobacter* strains harbouring the *ciaB* or *cadF* gene can adhere or invade intestinal cells. Some invasive and adherence factors other than those coded by the *ciaB* and *cadF* genes have been reported on *Campylobacter* surfaces [10], and this could have been the case in the current study population. Similar to our study, a lower prevalence of 76.4% in *ciaB* and 63.9% in *cadF* has been reported in Qatar, 66.7% and 51.5% in the Arabian Peninsula and 71.4% and 52.4% in Asia [11]. Most of the *cadF* genes in our study were expressed from the samples of children with diarrhoea (80.9%) and fever (69%).

Virulence genes were also expressed among the antibiotic susceptible isolates more than in the resistant isolates. These results corroborate the report of Al-Mahmeed et al. [100], and Rozynek et al. [101], where the adhesin genes tested were significantly associated with the antibiotic-susceptible strains. It, therefore, means that although a particular *Campylobacter* strain may be susceptible to a range of antibiotics, it may harbour virulence genes allowing it to elicit an infection of the same magnitude as a strain bearing a resistance gene. Studies have shown that there is a positive relationship between multi-virulence genes and the severity and duration of clinical symptoms [15].

### 4.6. Genetic Relatedness of Campylobacter Isolates from Human and Water Samples

A Newick tree was constructed to determine the genetic relatedness between *Campylobacter* isolates from water and stool. The tree revealed eight different groups in which, Groups II, V and VIII were *Campylobacter* strains that were exclusively circulating in paediatric populations, while groups IV were only found in water samples. These groups did not suggest any *Campylobacter* movement from human to water or vice versa. Four groups (I, II, VI and VII) were intermixed with *Campylobacter* strains isolated from both water and paediatric samples. These results show that these strains were closely related and may belong to the same lineage, suggesting that there was a possible transmission of *Campylobacter* infection from water to humans within the study population. These results support the notion that water is a significant source of human *Campylobacter* infections [53,102]. Seeing that strains from water and strains from humans shared the same group, it could be assumed that human isolates most likely originated from the water. Although *Campylobacter* species are known to be transmitted through the consumption of contaminated water, further studies involving a larger number of water samples would be needed to establish the association between stored household drinking water and the transmission of *Campylobacter* infections in the study area.

## 5. Conclusions

Control and prevention of campylobacteriosis in humans requires knowledge of transmission routes, antibiotic resistance profiles and virulence capacities of isolates. The results obtained in the present study showed the presence of three *Campylobacter* species in the studied communities displaying varying degrees of resistance, especially to the empirical drugs used for the treatment of *Campylobacter* infections. Also, while strains isolated in the study carried virulence genes, the detection rate of these genes was higher in the water samples than in the clinical isolates. Phylogenetic analysis revealed that *Campylobacter* infection from the studied communities might have been acquired through the consumption of contaminated water. It is, therefore, necessary to undertake continuous monitoring of the prevalence of *Campylobacter* and its associated virulence genes and antibiotic resistance profile to inform effective treatment regimens for *Campylobacter* infections. Finally, it is very important to emphasise that the presence of virulence genes is indicative and may not predict precisely how virulent a *Campylobacter* strain might be. Also, a negative result by real-time-PCR might not necessarily mean the absence of a gene but could be attributed to the sequence variation at the primer binding site or existence of another gene with a similar role.

## Figures and Tables

**Table 1:** Primers used for virulence and resistance genes.

Target Gene	Primer Name	Sequence (5′–3′)	Size (bp)	Reference
*cadF*	cadF-F2B cadF-R1B	CTAATACCTAAAGTTGAAAC CTAATACCTAAAGTTGAAAC	400	[42]
*ciaB*	ciaB-652 ciaB-1159	TGCGAGATTTTTCGAGAATG TGCCCGCCTTAGAACTTACA	527	[43]
*gryA*	GyrAF1 GyrAR1	CAACTGGTTCTAGCCTTTTG AATTTCACTCATAGCCTCACG	210	[40]
*tetO*	TetO	GTGACATCTTTTCAGTGGGAGG CTTCCATCTGCACATTCCCC	1014	[14]
*23S rRNA* at position 2074	23SRNA-F ERY2074R	TTAGCTAATGTTGCCCGTACCG TAGTAAAGGTCCACGGGGTCGC	486	[41]
*23S rRNA* at position 2074	23SRNA-F ERY2074R	TTAGCTAATGTTGCCCGTACCG AGTAAAGGTCCACGGGGTCTCG	485	[41]

**Table 2:** Distribution of *Campylobacter* spp. based on water source and point of collection.

Water Source	No. of Samples Collected	No. of *Campylobacter* Identified	*C. jejuni*	*C. coli*	*C. upsaliensis*
Direct Tap water	8	0	0	0	0
Stored Tap water	38	5 (13.2%)	2 (10%)	2 (10%)	1 (5%)
Stored well water	42	15 (35.7%)	9 (45%)	6 (30%)	0
River water	4	0	0	0	0
Total	92	20 (21.7%)	11 (55%)	8 (40%)	1 (5%)

**Table 3:** Antimicrobial resistance profile of human *Campylobacter* isolates.

Class of Antibiotic	Antibiotics	Code	Conc. (µg)	No. Resistant (%)	
				Human Samples	WATER Samples
Macrolides	Clarithromycin	CLR	15	44 (29.3)	19 (95)
	Erythromycin	ERY	15	40 (26.7)	17 (85)
Carbapenem	Meropenem	MEM	10	29 (19.3)	3 (15)
	Imipenem	IPM	10	23 (15.3)	0
β-lactam/β-lactamase inhibitor combination	Amoxicillin/clavulanic acid	AMX	30	97 (64.7)	6 (30)
Penicillin	Ampicillin	AMP	2	91 (60.7)	14 (70)
Fluoroquinolones	Ciprofloxacin	CIP	5	27 (18)	5 (25)
	Norfloxacin	NOR	10	17 (13.3)	8 (40)
Aminoglycosides	Amikacin	AMK	30	27 (18)	8 (40)
	Gentamicin	GEN	10	23 (15.3)	9 (45)
Tetracycline	Tetracycline	TET	30	48 (32)	11 (55)
	Tigecycline	TGC	15	45 (30)	9 (45)
Cephalosporine	Cephazolin	CFZ	30	90 (60)	10 (50)
	Cefuroxime	CXM	30	81 (54)	7 (35)

Note: EUCAST interpretation criteria for erythromycin on *C. jejuni* (˂20) and *C. coli* (˂24) was used in interpreting results for the macrolides. CLSI breakpoint for *Enterobacteriaceae* was used for aminoglycosides, carbapenems and fluoroquinolones.

**Table 4:** Antimicrobial resistance rates of *C. jejuni, C. coli* and *C. upsaliensis* from clinical specimens.

Antibiotics	*C. jejuni*		*C. coli*		*C. upsaliensis*	
	Human Samples (*n* = 66)	Water Samples (*n* = 11)	Human Samples (*n* = 59)	Water Samples (*n* = 8)	Human Samples (*n* = 25)	Water Samples (*n* = 1)
Clarithromycin	19 (28.7%)	10 (90.9%)	21 (35.5%)	8 (100%)	4 (16%)	0
Erythromycin	15 (22.7%)	11 (100%)	21 (35.5%)	6 (75%)	4 (16%)	0
Meropenem	9 (13.6%)	1 (9%)	16 (27%)	2 (25%)	4 (16%)	0
Imipenem	8 (12%)	0	13 (22%)	0	2 (8%)	0
Amoxicillin/clavulanic acid	44 (66.6%)	4 (36.4%)	36 (61%)	2 (25%)	17 (68%)	0
Ampicillin	40 (60.6%)	10 (90.9%)	37 (62.7%)	5 (62.5%)	14 (68%)	1 (100%)
Ciprofloxacin	16 (24.2%)	1 (9%)	11 (18.6%)	3 (37.5%)	0	1 (100%)
Norfloxacin	11 (16.6%)	2 (18%)	5 (8.4%)	5 (62.5%)	1 (4%)	1 (100%)
Amikacin	16 (24.2%)	4 (36.4%)	10 (16.9%)	3 (37.5%)	1 (4%)	1 (100%)
Gentamicin	14 (21.2%)	4 (36.4%)	9 (15.2%)	4 (50%)	0	1 (100%)
Tetracycline	24 (36.3%)	3 (27.3%)	19 (32.2%)	6 (75%)	5 (20%)	1 (100%)
Tigecycline	24 (36.3%)	3 (27.3%)	16 (32.2%)	6 (75%)	5 (20%)	1 (100%)
Cephazolin	41 (62%)	5 (45.5%)	35 (59.3%)	4 (50%)	14 (56%)	1 (100%)
Cefuroxime	33 (50%)	2 (18%)	37 (62.7%)	4 (50%)	11 (44%)	1 (100%)

**Table 5:** Distribution of MIC amongst the clinical *Campylobacter* isolates.

Antibiotics/MIC	*C. jejuni*		*C. coli*		*C. upsaliensis*	
	Human	Water	Human	Water	Human	Water
Erythromycin	16 (24.4%)	10 (90%)	22 (37.2%)	5 (62.5%)	4 (16%)	0
Ciprofloxacin	11 (16.6%)	1 (9%)	10 (16.9%)	3 (37.5%)	0	1 (100%)
Tetracycline	19 (28%)	3 (27.3%)	11 (18.6%)	6 (75%)	4 (16%)	1 (100%)
Ampicillin	37 (56%)	10 (90.9%)	29 (49%)	5 (62.5%)	14 (56%)	1 (100%)
Gentamicin	14 (21.2%)	4 (36.45)	6 (10%)	4 (50%)	0	1 (100%)

**Table 6:** Distribution of antibiotic resistance genes in *Campylobacter* isolates.

Species	*n*	Human Samples			Water Sample			
		*gryA* (%)	*tetO* (%)	Mutation at A2074C/A2075G (%)	*n*	*gryA* Gene (%)	*tetO* Gene (%)	Mutation at A2074C/A2075G (%)
*C. jejuni*	91	18 (19.7%)	29 (31.8)	17 (18.6)	11	1 (9)	3 (27.3)	8 (72.7)
*C. coli*	81	14 (17.2)	25 (30.8)	20 (24.6)	8	3 (37)	5 (62.5)	6 (75)
*C. upsaliensis*	34	6 (17.6)	5 (14.7)	3 (8.8)	1	1 (100)	0	1 (100)
Total 206	206	38 (18.4)	59 (28.6)	40(19.4)	20	5 (25)	8 (40)	15 (75)

**Table 7:** Distribution of virulence genes according to *Campylobacter* species.

*Campylobacter* spp.	Human Samples			Water Samples		
	*n*	*ciaB* (%)	*cadF* (%)	*n*	*ciaB* (%)	*cadF* (%)
*C. jejuni*	108	40 (37)	59 (54.6)	11	8 (72.7)	10 (90.9)
*C. coli*	89	35 (39.3)	48 (53.9)	8	7 (87.5)	6 (75)
*C. upsaliensis*	40	15 (37.5)	14 (35)	1	1(100)	1 (100)
Total	237	90 (38)	121 (51)	20	16 (80)	17 (85)
